# Bilateral Displacement of the Common Carotid Arteries by a Large Goiter

**DOI:** 10.7759/cureus.3298

**Published:** 2018-09-13

**Authors:** Lexian J McBain, Joe Iwanaga, Rod J Oskouian, Marios Loukas, R. Shane Tubbs

**Affiliations:** 1 Anatomical Sciences, St. George's University, St. George's, GRD; 2 Medical Education and Simulation, Seattle Science Foundation, Seattle, USA; 3 Neurosurgery, Swedish Neuroscience Institute, Seattle, USA; 4 Neurosurgery, Seattle Science Foundation, Seattle, USA

**Keywords:** goiter, thyroid gland, common carotid artery, cadaver, anatomy

## Abstract

Large goiters are less common in the developing world due to the use of iodized salt. However, these are seen occasionally. Herein, we report a case of very large goiter identified in a cadaver. This case is also of interest due to the significant lateral displacement of the common carotid arteries (CCA) and the midline shift of the trachea. This case and the salient literature addressing this topic have been discussed.

## Introduction

The thyroid gland is considered enlarged if its volume is greater than the normal range—17.9 to 145.0 cm^3^ [1]. In elderly patients, the position of the thyroid gland tends to be more caudal. [2]. Thyroid enlargement has several etiologies, including benign thyroid masses, malignant tumors and diffuse thyroid enlargement [3]. The characteristics of this enlargement vary from nodular to uniform. Multinodular goiters have been found to be the commonest cause of diffuse asymmetric enlargement of the thyroid gland [3]. In this case report, we examine thyroid enlargement, resulting from a goiter, and its effect on the common carotid artery (CCA) lying in close proximity to the gland [3].

## Case presentation

**Figure 1:**
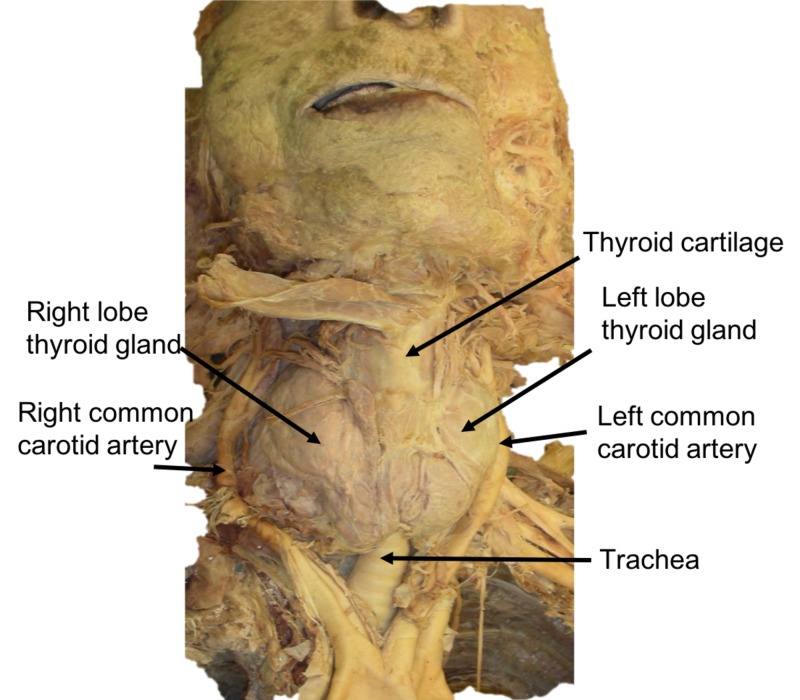
Anterior view of the case reported herein. Note the massively enlarged thyroid gland. Also observe the deviated left and right common carotid arteries and that the trachea is shifted to the left side of the specimen.

During the routine dissection of a female cadaver aged 89 years at death, a large mass was noted in the anterolateral neck. The cause of death was myocardial infarction. With further dissection, it was noted that the mass was, in fact, an enlarged thyroid gland with the right lobe found to be larger than the left (Figure 1). There were no signs of malignancy, and thus, the diagnosis of goiter was made. The thyroid was determined to be 8-10x its normal size, slightly on the left lobe and significantly on the right, causing distortion of the CCAs. In addition, the trachea was deviated to the left due to the enlarged thyroid gland. No other anatomical variations were noted in the head and neck region of this specimen. No additional pathologies were found in this specimen with further dissection of the neck.

## Discussion

An increase in the thyroid volume by 1.4 times has been known to cause CCA stenosis [1]. These patients tend to have symptoms of carotid ischemia. According to the World Health Organization (WHO), a goitrous thyroid gland would be one in which “each lateral lobe has a volume greater than the terminal phalanx of the thumbs of the subject being examined" [4]. Furthermore, the WHO has classified goiters into two grades. A grade one goiter is palpable but cannot be visualized when the neck is in the normal position. A grade two goiter is visible when the neck is in the normal position. The thyroid gland always appears enlarged in a grade two goiter [4].

Various structures adjacent to the thyroid gland

The thyroid gland is in close proximity to other structures in the neck. Close to the posterior and inner surface of the lateral lobes of the thyroid are the cricoid and thyroid cartilages, the inferior constrictor muscle of the pharynx, the inferior laryngeal nerve and other larger structures such as the esophagus and the trachea. On the anterior surface of the lateral lobes are the superficial muscles of the neck – sternohyoid, sternothyroid and omohyoid – and a small portion of the sternocleidomastoid muscle. Posterior to the outer portion of the lateral lobes is the carotid sheath containing the CCA, internal jugular vein (IJV) and the vagus nerve. The sternocleidomastoid muscle covers each lobe and hence guides the enlargement of the lateral lobes of the thyroid gland [5].

Various structures compressed by the goiters

The CCA is displaced posteriorly and laterally in cases of large goiters. The CCA can also be seen in the posterior triangle of the neck if the goiter is large or close to the anterior borders of the trapezius. Once displacement occurs, the carotid vessel may appear enlarged and tortuous, palpation of the vessel is felt as a tense subcutaneous cord [6]. The posterolateral displacement of the CCA due to the contraction of the vessel when the thyroid gland is inflamed is different from the displacement that occurs due to a goiter.

Upward growth of a goiter occurs within an angle formed by the CCA and the superior thyroid artery. As the goiter continues to enlarge, it hooks the carotid artery posterolateral, while the superior thyroid artery that is fixed by its terminal branches to the thyroid at the superior poles of the lateral lobe [5] holds the carotid artery in place, causing it to elongate as it is stretched. Crotti describes it as someone carrying another person on their shoulder swung across their neck. The displacement is dependent on the enlargement of the goiter, and there has been a report of a case of a 6-cm segment of a circle formed by the carotid artery around the goiter [7]. According to Hayashi [8], the fusion of the visceral fascia with the carotid sheath was often evident at the thyroid gland level, and Iwanaga et al. [6] hypothesized that fixed CCA by the visceral fascia does not curve at the thyroid gland level by itself. However, they did not mention the tortuous CCA caused by the goiter.

The posterolateral surface of the goiter becomes deeply grooved by the CCA. Additionally, a large separation is established between the CCA and the IJV due to the anatomical differences in the attachments of both CCA and IJV to the goiter. The artery is not tethered to the gland because there is no direct branch from the CCA to the thyroid gland, and therefore, the artery is free to move around when the gland enlarges. Posterior displacement of the artery, however, is limited by the spine [9].

A lateral displacement of the CCA is also significant for the presence of a goiter. In other pathologies such as malignancies that lead to cervical lymph node enlargement, the CCA is found in the center of the tumor. This occurs because the vascular cords of the neck are surrounded by the lymph nodes [10].

The patients present with a mass effect due to compression of the surrounding structures. In a case report done on a patient with a nodular goiter, it was found that in patients with a thyroid volume ranging from 102.9 to 145 cm^3^, the CCA was displaced outward with stenosis of the vessel and no direct route. Direct compression of the carotid has known to cause recurrent hemiplegia and aphasia during extension of the neck. Other symptoms such as transient ischemic attacks have not been directly associated with compression of the carotid but instead, thyrocervical steal due to increase in the thyroid blood flow is a greater cause of ischemia [10].

There is also a phenomenon known as mediastinal aberrant goiters that are found in the mediastinum arising from the ectopic thyroid tissue. These goiters can grow into the anterior mediastinum—anterior to the recurrent laryngeal nerve and anterolateral to the trachea or the posterior mediastinum close to the carotid sheath; their posterior growth can also compress the CCA [11].

Other structures compressed by goiter enlargement

Large goiters can compress the IJV and the subclavian veins, resulting in cyanosis and edema of the face, neck and the upper limbs. If the intrathoracic goiter is large, then the mediastinal vessels can become engorged and present as varicosity; this presentation is described as a “medusa head” [9].

A growing goiter can cause continuous irritation of the upper airway, resulting in respiratory symptoms such as shortness of breath, hoarseness and cough. Symptoms can range from mild to severe depending on the compression, and therefore, patients can experience anything from breathlessness or stridor to hypoxia and heart failure [9].

The esophagus can also be compressed leading to dysphagia; a barium swallow done on a patient with a goiter may possibly show stenosis and displacement of the esophagus [9,12].

Congestion of the inferior thyroid vein can occur due to compression of the superior vena cava, and other branches of the superior vena cava that drain blood from the caval part of the esophagus can also be compressed. Varices of the esophagus may present as a result of the compression of the superior vena cava between the right atrium and the azygos vein, and the compression of the hemiazygos can transmit to the esophageal mediastinal veins [9].

There has been an occurrence of vocal cord palsy in a patient with a substernal goiter [7]. This has resulted from the compression of the recurrent laryngeal nerve. In a study done in Germany, preoperative prevalence of vocal cord palsy was 3% in individuals with benign thyroid goiters [13].

## Conclusions

Enlargement of the thyroid gland to greater than 1.4 times its size is seen with goiters. Structures surrounding the gland including the CCAs can be either compressed or displaced. In the case examined, the only structures affected were the common carotid and the trachea with an enlargement of 8-10 times the gland’s original size. Clinicians who treat patients with large goiters should be aware of such displacements in order to monitor the developing symptoms.
